# Conservative Management of Primary Thyroid Mucosa-Associated Lymphoid Tissue (MALT) Lymphoma: A Case Report and Review of Multidisciplinary Approach

**DOI:** 10.7759/cureus.99894

**Published:** 2025-12-22

**Authors:** Muhammad Zahlaka, Abdellatif Zhalka, Shadi Shinnawi, Udi Cinamon, Abraham Goldfarb, Daniel Gabis

**Affiliations:** 1 Otolaryngology-Head and Neck Surgery, Wolfson Medical Center, Holon, ISR; 2 Endocrinology, Emek Medical Center, Kafr Qara, ISR; 3 Otolaryngology-Head and Neck Surgery, University of California Davis Health System, Sacramento, USA

**Keywords:** conservative management, hashimoto thyroiditis, malt lymphoma, multidisciplinary approach, thyroid lymphoma

## Abstract

Primary thyroid mucosa-associated lymphoid tissue (MALT) lymphoma is a rare condition. It often progresses slowly and has subtle symptoms. This condition is usually linked with chronic thyroiditis.

We present the case of a 70-year-old man who experienced progressive dysphagia and neck swelling for six months. He was found to have a large multinodular goitre and background hypothyroidism. Despite a fine-needle aspiration that did not provide a diagnosis, doctors decided to perform surgery because of his compressive symptoms. Histopathology showed extranodal marginal zone MALT lymphoma alongside chronic lymphocytic thyroiditis. Postoperative PET/CT and bone marrow tests revealed no signs of systemic involvement. After a review by a multidisciplinary tumor board, the patient was treated conservatively without additional therapy. He showed no signs of recurrence during the one-year follow-up.

This case highlights the diagnostic challenges and underscores the importance of considering MALT lymphoma when evaluating goitre. It also demonstrates that individualized, conservative management guided by a multidisciplinary team can lead to excellent outcomes in localized disease.

## Introduction

Papillary carcinoma is the most common type of thyroid cancer and has been extensively studied in clinical practice [1,2]. In contrast, primary lymphoma of the thyroid gland is much rarer, making up only 0.5% to 5% of all thyroid cancers and 2% to 7% of all extranodal lymphomas [3,4]. Most primary thyroid lymphomas (PTLs) are diffuse large B-cell lymphomas, which account for 60% to 80% of cases. Around 30% are classified as extranodal marginal zone lymphomas of mucosa-associated lymphoid tissue (MALT) type [5]. Primary thyroid MALT lymphoma is particularly rare, comprising 6% to 28% of all PTLs [4].

MALT lymphomas, in general, represent about one-quarter of all primary lymphomas. These lesions most often develop in the stomach (60% to 70%) but can also occur in other areas like the orbit, salivary glands, lungs, intestines, liver, and very rarely in the thyroid gland [6]. The cause of primary thyroid MALT lymphoma is thought to be closely linked to chronic lymphocytic thyroiditis, especially Hashimoto's thyroiditis, which creates ongoing antigen stimulation and lymphoid growth within the gland [3,4]. However, due to its rarity and often mild clinical signs, getting an early and correct diagnosis is a challenge [5]. It requires a high level of clinical awareness and a team-based approach.

Thyroid MALT lymphoma is known for its slow-growing nature compared to other thyroid cancers [5]. The disease usually progresses slowly and may not show "B" symptoms or clear systemic involvement, which makes it hard to distinguish from benign thyroid conditions based solely on clinical findings [1]. Its rarity, the complexities involved in diagnosis, and the lack of standard treatment guidelines due to limited data from randomized trials complicate management [7]. This reinforces the need for personalized care strategies.

This report discusses a rare case of primary thyroid MALT lymphoma, examining the clinical, radiological, and histopathological features. It emphasizes the importance of teamwork in both diagnosing and treating this unusual thyroid tumor.

## Case presentation

A 70-year-old man presented to our clinic in April 2024 with a six-month history of worsening difficulty swallowing and swelling in the central neck area. He also noticed losing 10 kilograms without trying, but denied having fever, night sweats, or other typical symptoms. His medical history included hypothyroidism, which he managed well with stable treatment, and polycythemia vera.

During the physical exam, he had a noticeable, bilateral multinodular goitre in the front of his neck, with no swollen lymph nodes felt in the neck. Lab tests showed hemoglobin at 16.7 g/dL (14-18 g/dL), white blood cell count at 12.7×10^3^/µL (4.5-11×10^3^/µL), lymphocytes at 2.3×10^3^/µL (1-4.8×10^3^/µL), TSH at 1.9 µU/mL (0.5-5 µU/mL), and free T4 at 11.5 pmol/L (10.3-24.5 pmol/L). Thyroid autoantibodies were not elevated.

**Figure 1:**
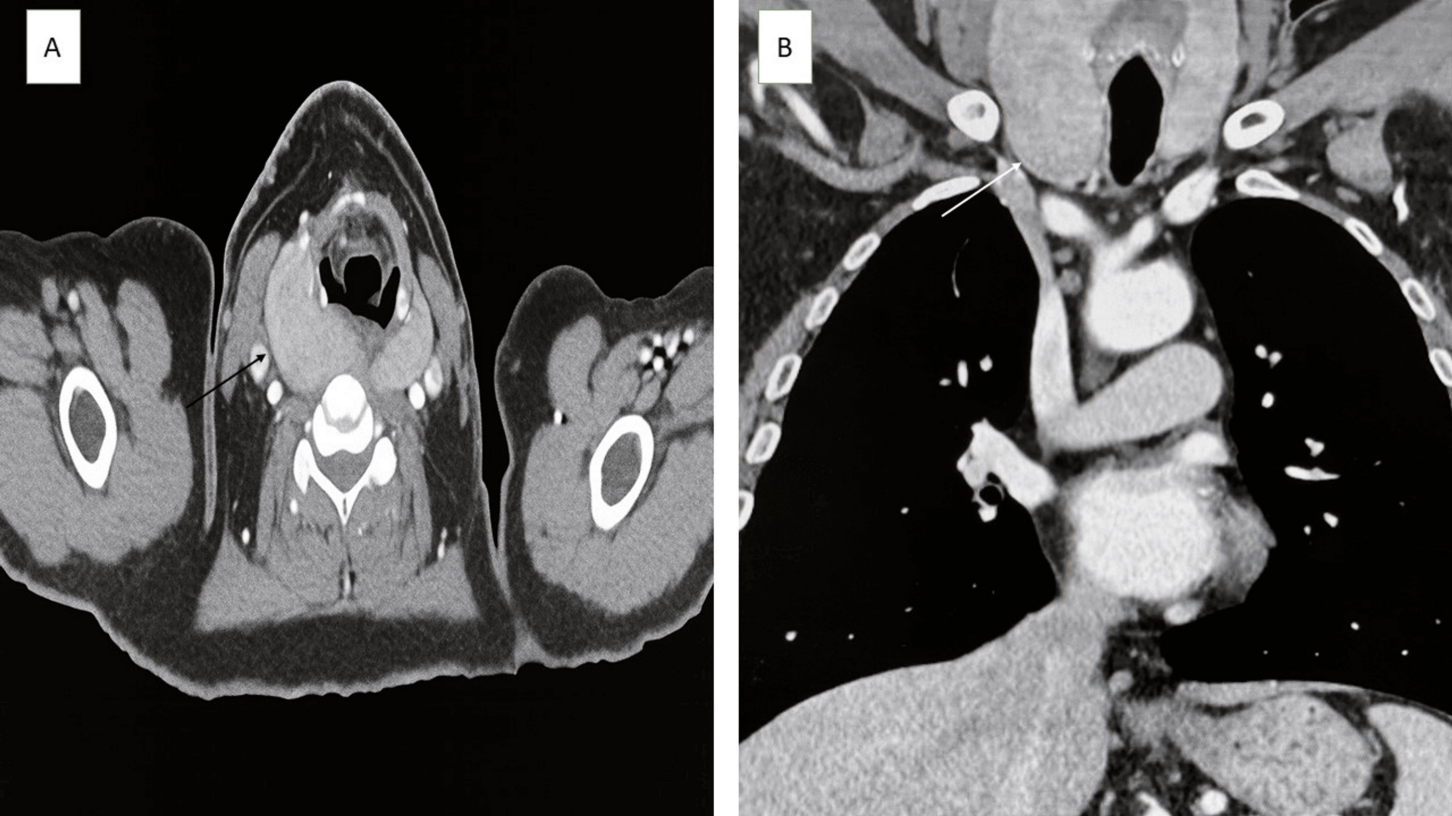
A. Axial CT showing diffusely enlarged thyroid gland (black arrow). B. Coronal CT showing enormous thyroid gland extending to the upper mediastinum (white arrow).

A neck ultrasound showed a very hypoechoic, irregular, multinodular goitre. The right lobe of the thyroid measured 10.5×6.5×6 cm, and the left lobe measured 8×3.2×3.2 cm, with no intrathyroid nodules found. A contrast-enhanced CT scan of the neck and chest confirmed an enlarged thyroid gland pressing on the esophagus and extending into the upper mediastinum, with no abnormal lymph nodes or signs of local invasion (Figure 1). A barium swallow test showed mild external pressure at the level of C6-C7, matching the swallowing difficulty. A fine-needle aspiration cytology done elsewhere did not provide a diagnosis.

Due to the goitre’s symptoms and concerns about cancer or worsening obstruction, the patient was sent to endocrine surgery. He had a near-total thyroidectomy without immediate surgical issues. No clear signs of cancer or lymphoma were seen during the operation, and no frozen section was done. The removed tissue was sent for a histopathological exam.

**Figure 2:**
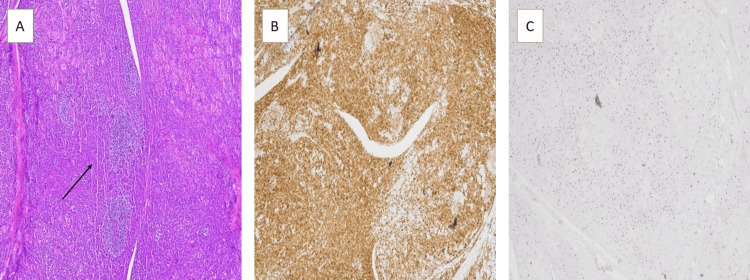
A. Histopathological examination shows lymphoepithelial lesions and low-grade B-cell lymphoma, H&E stain (black arrow). B. Positive for CD79a immunohistochemical stain. C. Positive for Ki67 immunohistochemical staining.

The pathological analysis showed extranodal marginal zone lymphoma of MALT, identified by lymphoepithelial lesions and chronic lymphocytic (Hashimoto) thyroiditis in the background, positive for CD79a and Ki67 immunohistochemical staining (Figure 2).

**Figure 3:**
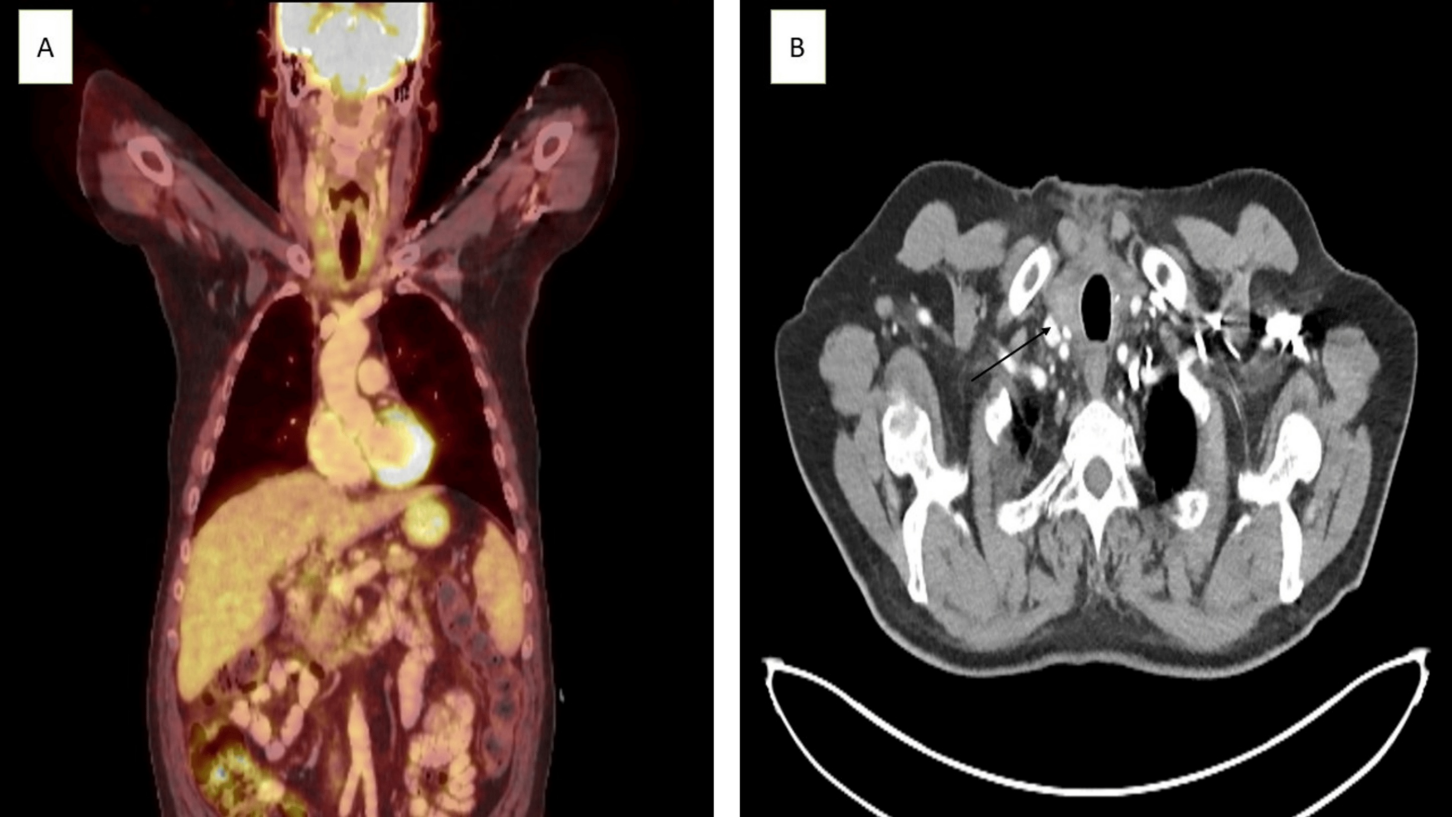
A. Six weeks post operative PET-CT showing no pathological uptake in the thyroid gland, neck lymph nodes, chest or abdomen. B. Post operative axial CT showing the thyroid gland remnants (black arrow).

A postoperative PET/CT scan showed no unusual FDG uptake in the thyroid area, cervical lymph nodes, chest, or abdomen (Figure 3), and a bone marrow exam was negative for lymphoma.

After a review by the tumor board, the decision was made that no further treatment was needed. The patient continued strict monitoring, and after one year of follow-up, he remains symptom-free, with no signs of local recurrence or systemic disease.

## Discussion

Primary thyroid MALT lymphoma is a rare clinical condition. It often has a slow progression and presents with mild, non-specific symptoms. Unlike most systemic lymphomas, only about 10% of patients with primary thyroid MALT lymphoma show "B" symptoms like fever, night sweats, and weight loss [5,7]. A similar percentage exhibit signs of hypothyroidism, often linked to Hashimoto’s thyroiditis, as seen in this case [8]. This underscores the important role of chronic autoimmune inflammation in the development of this lymphoma type [5].

Epidemiologically, the disease typically affects women in their 60s. When it occurs in men, the prognosis is usually less favorable compared to well-differentiated thyroid carcinoma [6,9-11]. Diagnostic challenges are significant. The overlapping features between cytological findings and benign thyroiditis may lead to inconclusive fine needle aspiration (FNA) results. In such cases, a core or surgical biopsy may be necessary for a definitive diagnosis, especially when clinical suspicion remains strong.

The management of thyroid MALT lymphoma is still debated. This is due to the lack of randomized controlled trials and the rarity of the disease. Retrospective reports highlight its slow behavior and favorable prognosis, supporting the use of organ-preserving treatments [5]. The commonly accepted approach recommends surgery for localized cases, while radiotherapy and chemotherapy are reserved for advanced or disseminated stages [12]. Recent studies promote radiotherapy as the primary treatment for stage I extranodal (IE) or stage II extranodal (IIE) disease, with adjuvant therapy considered only if there is suspected residual tumor after thyroidectomy [5,13]. In our case, even with unfavorable prognostic factors, surgery alone provided long-term disease control for over a year, showing that conservative management can be effective in strictly localized cases.

Recent molecular studies have started to identify genetic changes, like those in *TET2 *and* TNFRSF14*, that may explain the biology of thyroid MALT lymphoma. These findings could help with future risk assessment [14]. New therapies for marginal zone lymphoma, including monoclonal antibodies, targeted therapies, and cellular immunotherapy, are mostly experimental for localized thyroid cases, but they point toward more personalized medicine strategies [15].

## Conclusions

This case shows the need to think about MALT lymphoma when diagnosing progressive goiter, especially in patients with chronic thyroiditis. Personalized care that combines careful surgery with thorough follow-up can lead to great results in the early stages of the disease. Continued research in molecular profiling and long-term monitoring will be essential for improving treatment approaches and outcomes for patients with this rare lymphoma in the future.
